# Effect of timed dosing of usual antihypertensives according to patient chronotype on cardiovascular outcomes: the Chronotype sub-study cohort of the Treatment in Morning versus Evening (TIME) study

**DOI:** 10.1016/j.eclinm.2024.102633

**Published:** 2024-05-14

**Authors:** Filippo Pigazzani, Kenneth A. Dyar, Steve V. Morant, Céline Vetter, Amy Rogers, Robert W.V. Flynn, David A. Rorie, Isla S. Mackenzie, Francesco P. Cappuccio, Roberto Manfredini, Thomas M. MacDonald

**Affiliations:** a MEMO Research, Division of Molecular and Clinical Medicine, University of Dundee, UK; b Metabolic Physiology, Institute for Diabetes and Cancer, Helmholtz Munich, German Research Center for Environmental Health, and German Center for Diabetes Research (DZD), 85764 Neuherberg, Germany; c University of Colorado at Boulder, Colorado, USA; d University of Warwick, Warwick Medical School, Sleep Health & Society Programme, Coventry, UK; e University Strategic Center for Studies on Gender Medicine, Department of Medical Sciences, University of Ferrara, Ferrara, Italy

**Keywords:** Personalised chronotherapy, Hypertension, Chronotype, Cardiovascular outcomes, Dosing time, Antihypertensive

## Abstract

**Background:**

Timing drug administration to endogenous circadian rhythms may enhance treatment efficacy. In the Chronotype sub-study of the Treatment in Morning versus Evening (TIME) clinical trial we examined whether timing of usual antihypertensive medications according to patient chronotype (a behavioural marker of personal circadian rhythm) may influence clinical cardiovascular outcomes.

**Methods:**

This was a cohort sub-study of TIME, a prospective, randomised, open-label, blinded-endpoint, UK clinical trial of morning versus evening dosing of usual antihypertensive medications and cardiovascular outcomes. On August 3rd, 2020, all active TIME participants were invited to complete a validated chronotype questionnaire. Chronotype was quantitatively assessed as the mid sleep time on free days corrected for sleep debt on workdays (MSFsc). We analysed associations between chronotype and antihypertensive dosing time and explored their combined effect on cardiovascular outcomes (a composite endpoint of hospitalisation for non-fatal myocardial infarction (MI) or non-fatal stroke, and single components) using proportional hazard time-to-event models adjusted for baseline covariates. These were used to specifically test for interactions between dosing time and chronotype.

**Findings:**

Between August 3, 2020, and March 31, 2021, 5358 TIME participants completed the online questionnaire. 2778 were previously randomised to morning dosing and 2580 to evening dosing of their usual antihypertensives. Chronotype was symmetrically distributed around a median MSFsc of 3:07 am. The composite endpoint increased for later MSFsc (later chronotype) dosed in the morning but not in those dosed in the evening (hazard ratios 1.46 [95% CI 1.14–1.86] and 0.96 [95% CI 0.70–1.30] per hour of MSFsc, respectively; interaction p = 0.036). Later chronotype was associated with increased risk of hospitalisation for non-fatal MI in the morning dosing group, and reduced risk in the evening dosing group (hazard ratios 1.62 [95% CI 1.18–2.22] and 0.66 [95% CI 0.44–1.00] per hour of MSFsc, respectively; interaction p < 0.001). No interaction between chronotype and antihypertensive dosing time was observed for stroke events.

**Interpretation:**

Alignment of dosing time of usual antihypertensives with personal chronotype could lower the incidence of non-fatal MI compared to a ‘misaligned’ dosing time regimen. Future studies are warranted to establish whether synchronizing administration time of antihypertensive therapy with individual chronotype reduces risk of MI.

**Funding:**

The TIME study was funded by the 10.13039/501100000274British Heart Foundation (CS/14/1/30659) with support from the British and Irish Hypertension Society.

**Research in context**

**Evidence before this study**

A 2023 systematic review and meta-analysis of 72 randomised clinical trials investigating optimal dosing time for antihypertensive medications highlighted conflicting results. For over a decade, prospective clinical studies (mainly performed in northwest Spain) have reported a substantial reduction in major cardiovascular events with bedtime dosing of antihypertensives compared to morning dosing. In contrast, in a large UK general population with hypertension, evening versus morning dosing of usual antihypertensives showed no difference in terms of major cardiovascular outcomes [n = 21,104, HR 0.95 (95% CI 0.83–1.10); p = 0.53]. Recently published 2023 European Society of Hypertension Guidelines for the management of arterial hypertension concluded that, while bedtime dosing may be considered in patients with documented high night-time blood pressure, antihypertensives should be taken at a time of day that is convenient for the patient, which will usually be during the morning due to better treatment adherence. However, evidence from other clinical areas, including oncology and rheumatology, suggests that selecting the administration time of drugs according to endogenous circadian rhythms (chronotherapy) may enhance treatment efficacy and reduce unwanted side effects. Humans show large inter-individual differences in their 24-h preference for sleep and wakefulness (morning vs evening). These rhythmic behaviours, termed chronotype, reflect complex interactions between genetically encoded circadian ‘clocks’ and personal phase entrainment to environmental cues linked to light/dark cycles. Previous studies on the chronotherapy of hypertension have not stratified participants according to chronotype, an established marker of personal circadian rhythm.

We designed the Chronotype sub-study of TIME to explore whether administration time of usual antihypertensives according to chronotype (personalized chronotherapy) might influence major cardiovascular clinical outcomes.

**Added value of this study**

The Chronotype sub-study was a prospective cohort study within the Treatment in Morning versus Evening (TIME) study and was designed to explore the combined effect of timing of usual antihypertensives and chronotype on major cardiovascular outcomes in adults with arterial hypertension. To our knowledge, this is the first study providing evidence of an interaction between administration time of usual antihypertensives and personal chronotype. We observed a lower rate of non-fatal myocardial infarction events when dosing time was synchronized with chronotype, specifically in later chronotypes receiving evening dosing of antihypertensives and in earlier chronotypes receiving morning dosing. In line with the TIME study results, we found no effect of dosing time on cardiovascular outcomes in intermediate chronotypes (∼50% of the study population). Additionally, we observed that later chronotypes showed a trend towards an increased risk of non-fatal stroke, independent of dosing time.

**Implications of all the available evidence**

These findings significantly complement the existing hypertension chronotherapy literature by highlighting the clinical importance of personal chronotype as a potential therapeutic target. Selection of the appropriate medication and adherence to treatment remain priorities in treating individuals with hypertension. However, physicians could quickly assess a patient's chronotype to better stratify patient risk and tailor the administration of antihypertensives, delivering morning dosing to morning patients (earlier chronotypes) and evening dosing to evening patients (later chronotypes) to provide additional cardiovascular protection. Further research is warranted to independently confirm our findings and determine whether a personalized chronotherapeutic approach can become a pragmatic and complementary strategy to improve the cardiovascular outlook of people with hypertension.

## Introduction

Hypertension is a leading worldwide modifiable risk factor for cardiovascular disease morbidity and mortality.^1^ Despite significant progress from evidence-based lifestyle modifications and drug therapies, strategies to achieve good blood pressure control and novel treatments remain an urgent public health need.^2^ More personalized approaches tailoring prevention and care to the individual are expected to emerge, but first require precise clinical phenotyping, including consideration of relevant genetic and environmental risks and interactions.^3^

Like most physiological functions, blood pressure (BP) is regulated by the circadian timing system according to a ∼24-h rhythm.^4^ Since the late 1990s, the circadian rhythm of BP, with a prominent peak in the morning after awakening and a secondary peak in the afternoon, has been considered among other possible circadian triggers of unfavourable cardiovascular events, including the assumption of upright posture, increased platelet aggregability, changes in blood clotting and fibrinolysis.^5^^,^^6^ Moreover, loss of diurnal BP rhythm, with an exaggerated rise in the morning (i.e., morning surge) and blunted dipping at night (i.e., nocturnal hypertension), are associated with an increased risk of adverse cardiovascular outcomes.^7^ Temporal patterns in cardiovascular events have given rise to a dynamic rather than static concept of risk, termed ‘chronorisk’.^8^ Indeed, morning hours are well-known periods of greatest risk for myocardial infarction, stroke, and rupture of aortic aneurysms.^9^, ^10^, ^11^

Increased awareness of the importance of circadian timing in determining health and disease^12^ has recently brought forth the concept of circadian medicine.^13^ Modern chronotherapy exemplifies this tailored pharmacological approach by delivering therapies to patients at times when they will be more effective and tolerable.^14^^,^^15^ Hence, bedtime dosing of antihypertensive medications has been proposed as a chronotherapeutic approach to control BP. Studies conducted in Spain reported that bedtime administration of antihypertensive medications reduced cardiovascular outcomes.^16^ However, other prospective clinical trials investigating the potential advantages of evening versus morning dosing of antihypertensive drugs have generated conflicting results.^17^ Recently, in our Treatment in Morning versus Evening (TIME) study of a general UK population with hypertension, we found that dosing time of usual antihypertensives did not affect major cardiovascular events.^18^ Thus, the 2023 European Society of Hypertension Guidelines for the management of arterial hypertension suggest to consider bedtime dosing in patients with documented nocturnal hypertension, but recommend taking antihypertensives in the morning due to better adherence.^19^

However, any attempt at personalized therapy cannot ignore that humans show wide inter-individual circadian differences –termed chronotype–in their preferences for sleep and wakefulness (i.e. early or morning vs late or evening) over 24 h. Earlier chronotypes (the proverbial “morning larks”) are individuals who rise earlier and show peak alertness in the mid-morning hours, whereas later chronotypes (“night owls”) are late risers who exhibit peak alertness later in the day, often late into the evening.^20^ Expression of chronotype, from early to late, is normally distributed in the population, but differences according to age and sex have been reported.^21^ The greatest changes in chronotype occur between age 15–25 for both sexes, after which chronotype becomes earlier and relatively stable at the individual level.^21^^,^^22^ These rhythmic physiological and behavioural manifestations reflect complex interactions between the genetically encoded circadian timing system and personal phase entrainment to environmental cues linked to light/dark cycles.^23^^,^^24^ Importantly, chronotype has been linked to health outcomes.^25^ Later chronotypes have an increased risk for cardiovascular disease and mortality when compared to earlier chronotypes.^26^ Later types tend to adopt more unhealthy lifestyle habits, and have unfavourable cardiovascular, and metabolic profiles,^27^^,^^28^ with additional sex-specific differences.^29^ Moreover, chronotype-dependent differences in the timing of acute cardiovascular events^30^ suggest the potential benefit of a more personalized chronotherapeutic approach to cardiovascular disease.

Evidence from randomised clinical trials in non-cardiovascular disease areas has reported a substantial benefit of chronotherapy, suggesting that synchronising drug administration with endogenous circadian rhythms can enhance treatment efficacy.^31^^,^^32^

Whether an individual's chronotype may modify the efficacy of antihypertensive medications has never been investigated, nor has whether timing antihypertensives according to patient chronotype can enhance their efficacy.

In the Chronotype sub-study cohort of the TIME study, we aimed to examine whether and to what extent timed administration of antihypertensives synchronized with a patient's chronotype (i.e. personalized chronotherapy) could maximise the beneficial effects of antihypertensive treatment in preventing major cardiovascular events.

## Methods

### Study design

This was a cohort sub-study of the Treatment in Morning versus Evening (TIME) study. TIME was a large, prospective, pragmatic, decentralised, parallel-group study which assessed, in more than 21,000 hypertensive adults in the UK who took their usual prescribed antihypertensive medications at a single time of day (excluding night shift workers), whether there are differences in major cardiovascular outcomes between those randomised to take their usual antihypertensive medications in the morning (06:00–10:00) or in the evening (20:00–00:00). The sub-study was designed and implemented during the TIME study follow-up period and before database lock. The objective of the Chronotype sub-study was reported in the TIME study protocol.^18^ This report follows the Strengthening the Reporting of Observational Studies in Epidemiology (STROBE) reporting guidelines. The TIME Chronotype sub-study was approved by the East of Scotland Research Ethics Committee (11/AL/0309), and the TIME steering committee approved the Chronotype sub-study on 25th November 2019. The TIME study was funded by the British Heart Foundation, supported by the British and Irish Hypertension Society, and The University of Dundee was the study Sponsor.

### Study participants

On August 3rd, 2020, all active participants in the TIME study were invited to take part in the Chronotype sub-study. After providing informed consent, participants completed an online questionnaire.

### Procedures

We analysed associations between chronotype and antihypertensive dosing time and explored their combined effect on cardiovascular outcomes.

### Chronotype

Individual chronotype was quantitatively assessed using the ultra-short version of the Munich ChronoType Questionnaire (μMCTQ)^33^ (Figure S1, Appendix p 16). This shortened version contains only the core chronotype module of the standard Munich ChronoType Questionnaire (stdMCTQ), and results obtained by the μMCTQ and the stdMCTQ are in good agreement with each other. The μMCTQ shows good test-retest reliability and has been validated with gold-standard circadian phase markers, including dim-light melatonin onset (DLMO) and actigraphy.^33^ We estimated chronotype as midpoint of sleep on free days (MSF) corrected for sleep debt on workdays (MSFsc) to remove the confounder of adaptive responses due to social schedules.^33^ Sleep midpoint (hours:minutes) was treated as a continuous variable, with later midpoints of sleep indicating a later chronotype and earlier midpoints of sleep an earlier chronotype.

For internal validation, we also assessed chronotype qualitatively by asking participants to self-report their preference for ‘morningness’ or ‘eveningness’ through question number 19 of the Morningness-Eveningness Questionnaire (MEQ) that defines four categories: “definitely a morning type”, “rather more a morning type than an evening type”, “rather more an evening type than a morning type”, “definitely an evening type” (coded 1 to 4 when treated as a continuous variable).^20^ The answer to this single question shows 89% correlation with the full MEQ classification and captures a substantial part of genetic variance associated with chronotype.^34^ Because of their brevity, the μMCTQ and the single-question MEQ are ideal tools to rapidly estimate chronotype in time- and resource-critical contexts, such as in large cohort studies focused on personalized medicine, like this Chronotype sub-study.

### Outcomes

The primary outcome of the Chronotype sub-study was modified from the primary outcome of the TIME study (composite of vascular death, hospitalisation for non-fatal MI or non-fatal stroke) because invitations to take part in the sub-study were issued only eight months before the TIME study closed and participants who had died before could not have completed it.

We used as the primary endpoint a composite of hospitalisation for non-fatal MI and non-fatal stroke, which were analysed as the time to first event from randomisation to the end of the TIME study. Secondary outcomes were hospitalisation for non-fatal MI, non-fatal stroke, congestive heart failure, pre-specified self-reported adverse events, non-adherence to allocated dosing time and self-reported home blood pressure. There were only few cases of congestive heart failure to analyse. Cardiovascular outcomes were predominantly non-fatal events that occurred before the sub-study questionnaire was completed, but subsequent events (n = 16) were also included and one of these was fatal (death for congestive heart failure). All potential cardiovascular endpoints in the TIME study were adjudicated by an independent clinical endpoint committee blinded to dosing time allocation. Pre-specified participant-reported adverse events and adherence to randomised dosing time were also assessed.

### Statistical analysis

The statistical analysis plan, available in the online supplement (Appendix p 21), specifies exploratory analyses of outcomes using Cox proportional-hazards models. Tests of the proportional hazards assumption were met. We therefore present hazard ratios for chronotype variables adjusted for sex, age, smoking status, history of heart attack or stroke, number of antihypertensive drugs and their mean half-life. We tested for interactions between dosing time and mid sleep time and self-reported chronotype, and we illustrated trends using categorised versions of these variables. Sensitivity analyses were also carried out on the on-treatment population for cardiovascular outcomes. Additionally, sex-based analyses were performed per the Sex and Gender Equity in Research (SAGER) guidelines.^35^ All analyses were conducted using R (version 4.1.1).^36^

### Role of the funding source

The British Heart Foundation funded the TIME study and had no role in this sub-study design, data collection, data analysis, data interpretation, or writing of the report. The corresponding authors had full access to data in the study and had final responsibility for the decision to submit for publication.

## Results

**Fig. 1:**
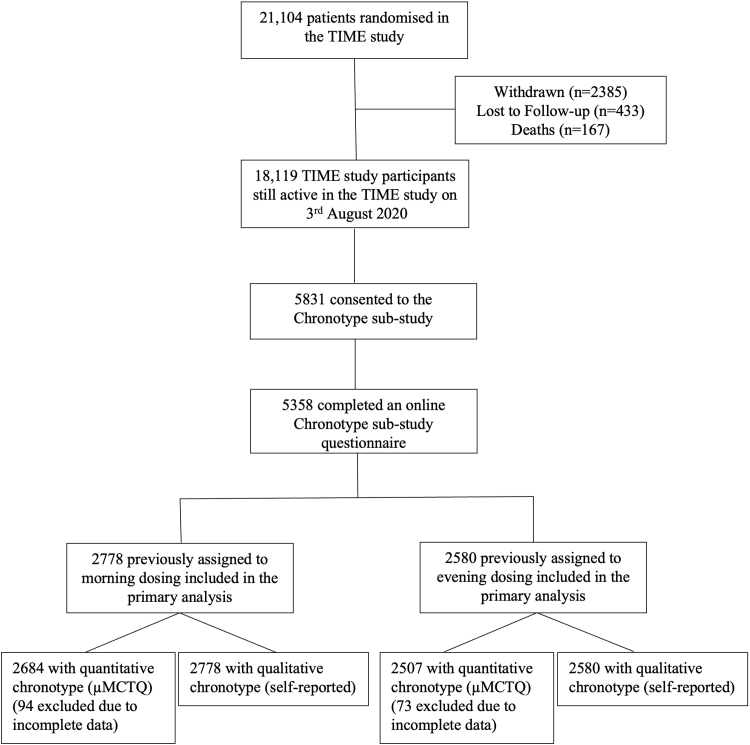
Study profile.

Between August 3, 2020, and March 31, 2021, 5831 of all active TIME study participants (18,119) consented to participate in the Chronotype sub-study. Among the 21,104 participants included in the TIME study primary analysis, 5358 (25.4%) completed an online questionnaire assessing chronotype; 2778 [51.8%] were previously randomised to the TIME morning dosing group and 2580 [48.2%] to the TIME evening dosing group. We were unable to calculate mid sleep times for 167 participants (94 [3.4%] in the morning group and 73 [2.8%] in the evening group) because data were incomplete, so we excluded them from further analyses (Fig. 1).

### Baseline characteristics

Baseline characteristics in the Chronotype sub-study were balanced between morning and evening dosing groups (Table 1) and did not differ substantially from those of the main TIME study population (Table S1; Appendix p 3). Mean age of participants at the sub-study entry was 64.4 years (SD 8.3); with 2292 [42.8%] females and 3066 [57.2%] males (Figure S2, Appendix p 17); 5092 (95.0%) were White; and 604 (11.3%) had previous cardiovascular disease. In the Chronotype sub-study cohort, participants were younger (64.4 years [SD 8.32] vs 65.3 years [SD 9.58]), less likely to have cardiovascular risk factors, and more likely to have reported details of their prescribed antihypertensives at the beginning of the TIME study than those in the main study population (Table S1, Appendix p 3 and Table S2a and b, Appendix p 5).

**Table 1:** Baseline characteristics for Chronotype sub-study.

Allocated dosing time	Morning	Evening
	(N = 2778)	(N = 2580)
Age, yrs		
Mean (SD)	64.4 (8.21)	64.5 (8.44)
Missing	10 (0.4%)	8 (0.3%)
Sex		
Female	1193 (42.9%)	1099 (42.6%)
Male	1585 (57.1%)	1481 (57.4%)
Country of residence		
Scotland	235 (8.5%)	214 (8.3%)
England	2447 (88.1%)	2265 (87.8%)
Wales	95 (3.4%)	101 (3.9%)
Ireland	1 (0.0%)	0 (0%)
Ethnicity		
Asian or Asian British	9 (0.3%)	8 (0.3%)
Black, African, Caribbean, or Black British	7 (0.3%)	7 (0.3%)
Multiple or mixed	13 (0.5%)	10 (0.4%)
Not reported	101 (3.6%)	99 (3.8%)
Other	7 (0.3%)	5 (0.2%)
White	2641 (95.1%)	2451 (95.0%)
Smoking status		
Never	1616 (58.2%)	1556 (60.3%)
Former	1037 (37.3%)	939 (36.4%)
Current	109 (3.9%)	77 (3.0%)
Missing	16 (0.6%)	8 (0.3%)
Systolic blood pressure, mmHg^d^		
n	1377	1387
Mean (SD)	135 (12.9)	134 (12.4)
Diastolic blood pressure, mmHg^d^		
n	1376	1385
Mean (SD)	78.8 (9.18)	79.1 (8.75)
Body-mass index, kg/m^2^^a^		
n	2623	2441
Mean (SD)	28.2 (4.76)	28.0 (4.80)
Cardiovascular history^b^		
Evidence of cardiovascular disease^c^	319 (11.5%)	285 (11.0%)
Previous myocardial infarction	99 (3.6%)	94 (3.6%)
Angina, requiring medical treatment	76 (2.7%)	60 (2.3%)
Previous stroke	54 (1.9%)	60 (2.3%)
Previous transient ischaemic attack	113 (4.1%)	103 (4.0%)
Peripheral vascular disease	35 (1.3%)	27 (1.0%)
Other medical history^b^		
Diabetes		
Yes, on medication	241 (8.7%)	218 (8.4%)
Yes, not on medication	85 (3.1%)	79 (3.1%)
Yes, medication unknown	1 (0.0%)	0 (0%)
Asthma		
Yes, on medication	242 (8.7%)	232 (9.0%)
Yes, not on medication	21 (0.8%)	14 (0.5%)
Kidney impairment		
Yes, on medication	7 (0.3%)	10 (0.4%)
Yes, not on medication	63 (2.3%)	79 (3.1%)
Chronic obstructive pulmonary disease		
Yes, on medication	50 (1.8%)	38 (1.5%)
Yes, not on medication	13 (0.5%)	11 (0.4%)
Arthritis, requiring medical treatment		
Yes, on medication	173 (6.2%)	132 (5.1%)
Yes, not on medication	91 (3.3%)	71 (2.8%)
Yes, medication unknown	0 (0%)	0 (0%)
Antihypertensive use at study entry^b^, number of medications		
Mean (SD)	1.52 (0.726)	1.48 (0.701)
Chronotype as mid sleep time on free days corrected for sleep debt on workdays (MSFsc)		
Median	3:07 am	3:07 am
‘earlier chronotype’ (>30mins before median)	662 (23.8%)	673 (26.1%)
‘intermediate chronotype’ (Within 30mins of median)	1272 (45.8%)	1170 (45.3%)
‘later chronotype’ (>30mins after median)	750 (27.0%)	664 (25.7%)
Self-reported chronotype		
Definitely a morning type	889 (32.0%)	780 (30.2%)
More morning than evening	974 (35.1%)	898 (34.8%)
More evening than morning	598 (21.5%)	589 (22.8%)
Definitely an evening type	317 (11.4%)	313 (12.1%)

Data are mean (SD) or n (%) unless otherwise stated.

a Derived from self-reported height and weight.

b Self-reported medical history.

c Defined as self-reported history of angina, myocardial infarction, stroke, transient ischaemic attack, or peripheral vascular disease.

d Self-reported last known measurement.

Mid sleep times corrected for sleep debt on workdays (MSFsc) were symmetrically distributed around a median of 3:07 am (Table 1 and Figure S3, Appendix p 18). 662 (23.8%) participants in the morning dosing group and 673 (26.1%) in the evening group had MSFsc >30 min before the median and were considered ‘earlier’ chronotype. 750 (27.0%) participants in the morning dosing group and 664 (25.7%) in the evening group had MSFsc >30 min after the median and were considered ‘later’ chronotype. 1272 (45.8%) participants of the morning dosing group and 1170 (45.3%) of the evening dosing group had MSFsc within 30 min of the median and were considered an ‘intermediate’ chronotype. Self-reported chronotype was balanced between morning and evening dosing groups (Table 1).

**Fig. 2:**
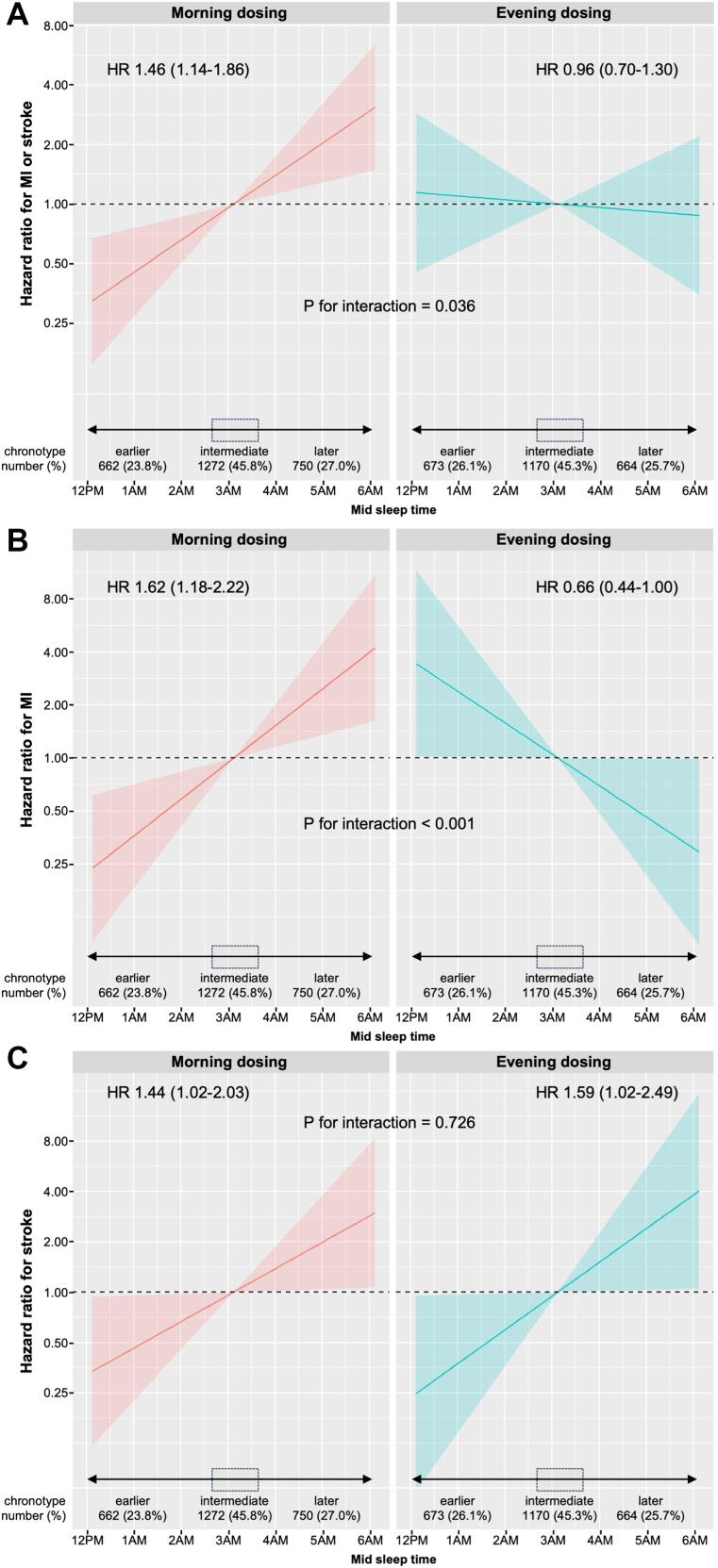
Hazard ratios (95% CI) for non-fatal cardiovascular outcomes vs mid sleep time (per hour advance in MSFsc). A) Composite endpoint of hospitalisation for non-fatal myocardial infarction (Ml) or non-fatal stroke, B) Hospitalisation for non-fatal myocardial infarction (MI), and C) Hospitalisation for non-fatal stroke. The interaction p-value tests whether the HR is different between morning and evening dosing. MSFsc, Mid sleep time on free days corrected for sleep debt on workdays (chronotype).

In the intention-to-treat analysis, the composite endpoint of hospitalisation for non-fatal MI and non-fatal stroke increased for later MSFsc (later chronotype) treated in the morning, but not in those treated in the evening (hazard ratios 1.46 [95% CI 1.14–1.86] and 0.96 [95% CI 0.70–1.30] per hour of MSFsc respectively; interaction p = 0.036; Table 2; Fig. 2A). Later chronotype was associated with an increased risk of hospitalisation for non-fatal myocardial infarction in the morning dosing group and reduced risk in the evening dosing group (hazard ratios 1.62 [95% CI 1.18–2.22] and 0.66 [95% CI 0.44–1.00] per hour of MSFsc respectively; interaction p < 0.001; Table 2; Fig. 2B). The risk of hospitalisation for non-fatal stroke increased with later chronotype in both dosing time groups (interaction p = 0.726; Table 2, Fig. 2C). For intermediate chronotypes (n = 2442 participants with MSFsc within 30 min of the median, 3:07 am), we found no effect of dosing time on cardiovascular outcomes (Fig. 2A–C; Figure S4, Appendix p 19 and Figure S5, Appendix p 20).

**Table 2:** Hazard ratio for non-fatal cardiovascular outcomes per hour of MSFsc^a^ in the intention-to-treat cohort.

Outcome	Dosing time	Unadjusted event rate			Linear trend	
		Patient years	Events	Event rate^b^	Hazard ratio^c^ (95% CI)	Interaction^d^
Composite of hospitalisation for non-fatal myocardial infarction or stroke	Morning	14,589	54	3.7	1.46 (1.14, 1.86)	p = 0.036
	Evening	13,532	46	3.4	0.96 (0.70, 1.30)	
Hospitalisation for non-fatal myocardial infarction	Morning	14,637	30	2.0	1.62 (1.18, 2.22)	p < 0.001
	Evening	13,588	26	1.9	0.66 (0.44, 1.00)	
Hospitalisation for non-fatal stroke	Morning	14,672	23	1.6	1.44 (1.02, 2.03)	p = 0.726
	Evening	13,608	19	1.4	1.59 (1.02, 2.49)	

Hazard ratio for non-fatal cardiovascular outcomes per hour advance in MSFsc in the intention-to-treat cohort. The interaction term tests whether the HR is different between morning and evening dosing.

a Mid sleep time on free days corrected for sleep debt on workdays (chronotype).

b Events per thousand patient years.

c Hazard ratio for each hour advance in MSFsc, estimated over an observed range of approximately 12 am (earliest chronotype) to 6 am (latest chronotype).

d Test for different trends with MSFsc on morning and evening dosing.

Using the score for self-reported chronotype led to similar results. The risk of non-fatal MI for later chronotypes increased in the morning dosing group and decreased in the evening dosing group (hazard ratios 1.27 [95% CI 0.89–1.80] and 0.58 [95% CI 0.36–0.91] per unit score respectively; interaction p = 0.006; Table S3, Appendix p 6). The risk of stroke was higher in later chronotypes in both dosing time groups (interaction p = 0.742).

Analyses of hospitalisations for heart failure lacked statistical power due to the small number of events observed, and no trends with chronotype variables were detected (Table S3, Appendix p 6).

The on-treatment sensitivity analysis included 4565 participants after the exclusion of 180 (6.5%) participants randomised to morning dosing, and 446 (17.3%) participants randomised to evening dosing who reported not dosing at their allocated time at the end of the TIME study. The on-treatment analysis confirmed the increased risk of hospitalisation for non-fatal MI in the morning dosing group and a reduced risk in the evening dosing group for those with either later MSFsc (hazard ratios 1.68 [95% CI 1.23–2.30] and 0.70 [95% CI 0.44–1.11] per hour of MSFsc respectively; interaction p = 0.002) or high score for self-reported chronotype (i.e. later chronotype; interaction p = 0.016; Table S4, Appendix p 7).

Risks of non-fatal MI and non-fatal stroke were higher in males than in females, except for a similar risk of stroke with morning dosing (Table S5, Appendix p 8). Because there was a strong interaction between dosing time and chronotype for non-fatal MI, interactions between sex and chronotype were estimated separately for morning and evening dosing for this outcome. There was no evidence that any association between outcomes and the chronotype variables differed between men and women. Analysis of self-reported adverse events showed no significant interaction between chronotype and dosing time (Table S6, Appendix p 9).

Non-adherence to randomised dosing time at any time occurred in 1702 (31.8%) participants in the Chronotype sub-study cohort compared to 4773 (30.3%) in the TIME study population. No association was found between chronotype and adherence to allocated dosing time in either group (Table S7, Appendix p11).

Of the 7657 TIME participants who reported owning a home blood BP monitor and submitted at least one set of measurements, 2452 (32.0%) consented to the Chronotype sub-study (1234 [32.1%] in the evening dosing group and 1218 [31.9%] in the morning dosing group). In the TIME study we observed that participants assigned to evening dosing had higher evening BP than those allocated to morning dosing. In the Chronotype cohort, we additionally found that later chronotype was associated with an increase in mean evening-assessed BP in the evening dosing group (systolic BP difference 0.87 [95% CI 0.25–1.49] mm Hg per hour of MSFsc), but not in the morning dosing group (systolic BP difference 0.26 [95% CI −0.35 to 0.87] mm Hg per hour of MSFsc; Table S8, Appendix p 12). No interaction between chronotype and diastolic home BP was found.

## Discussion

The results of the Chronotype sub-study of the TIME (Treatment In Morning versus Evening) large pragmatic clinical trial in patients with hypertension suggest that (a) later chronotypes (night owls) had a reduced risk of hospitalisation and incidence of non-fatal MI when taking antihypertensive medications in the evening rather than in the morning; (b) conversely, earlier chronotypes (morning larks) had a reduced risk of hospitalisation and incidence of non-fatal MI when taking antihypertensives in the morning rather than in the evening; (c) the risks of hospitalisations and incidence of stroke and congestive heart failure did not differ by dosing time when chronotype was considered; (d) in agreement with the main TIME result, intermediate chronotypes (around 50% of the sub-study population) showed no difference in cardiovascular outcomes between morning and evening dosing.

All humans have a genetically determined internal circadian timing system.^13^ Intracellular circadian ‘clocks’ control daily rhythms of cardiovascular physiology and pathophysiology and diurnal variation in response to therapies.^12^ Three decades ago, the possibility of a pharmacological treatment of cardiovascular diseases aligned with the circadian rhythms of the cardiovascular system was suggested.^37^ Since individual differences in circadian timing exist, and later chronotypes are exposed to a higher cardiovascular risk,^27^ personal chronotype and relative phase alignment are important clinical parameters to consider for each patient.

We found that the risk for non-fatal myocardial infarction (MI) was lower in later chronotypes dosing their usual antihypertensive medications in the evening, and in earlier chronotypes dosing their antihypertensives in the morning. Conversely, the risk of non-fatal stroke was not influenced by the interaction of chronotype with the administration time of antihypertensives, despite our observation that later chronotypes had a higher risk of stroke compared to earlier chronotypes, both in males and females.

These findings are a valuable refinement of the original TIME study results since they provide novel information regarding personalised medicine in the management of hypertension. Administration time of usual antihypertensives aligned to individual chronotype was associated with a lower risk of MI. Nevertheless, although acute cardiovascular events follow a circadian distribution,^5^^,^^7^ limited evidence is available on possible relationships with chronotype. Individual chronotype may influence vascular endothelial vasodilation and cardiovascular responses to stress.^38^^,^^39^ Moreover, there are chronotype-dependent differences in the incidence of MI, with a morning peak in earlier chronotypes and an afternoon peak for later chronotypes.^30^ This shift in internal circadian timing between morning and evening individuals could explain the different peak times in the onset of non-fatal MI events, and potentially justifies the cardiac benefit of an antihypertensive therapy synchronized with personal circadian rhythm.

In the Chronotype sub-study cohort later chronotypes had a more unfavourable cardiovascular and metabolic risk profile than earlier chronotypes (Table S9, Appendix p 13). Additionally, later chronotypes had a significant increase in mean evening-assessed systolic BP in the evening dosing group compared to the morning dosing group. Hence, it is possible that the greater cardioprotective effect of evening dosing in later chronotypes may be, at least in part, related to a more effective control of their higher nocturnal BP.^40^ However, as the TIME study did not collect nocturnal BP readings, this explanation remains speculative, since the vascular protection attributable to lower BP burden was not detected for stroke. On the other hand, a novel finding of our study is that morning dosing of antihypertensive medications in early chronotypes appears to confer better pharmacologic protection against cardiac ischemic risk. Thus, not only bedtime dosing, but also morning dosing could lower the incidence of non-fatal MI when dosing time of usual antihypertensives is aligned to personal endogenous rhythm.

The stroke subgroup analysis was limited by the small sample size, and certainly deserves further investigation. Nevertheless, we observed that later chronotypes showed an increased risk of non-fatal stroke, reinforcing the available literature suggesting evening chronotype as a potential risk factor for cardiovascular events.^27^^,^^41^

The Chronotype sub-study was planned in 2018, after the 2017 Nobel Prize for discoveries of molecular mechanisms controlling circadian rhythms,^42^ and implemented before database lock for the TIME study. The objective was to investigate associations, and to generate hypotheses for further investigation with independent prospective cohorts.

The study has some limitations to consider. The high ethnic homogeneity of participants limits the generalisability of our findings, as 95% of patients were White. The chronotype questionnaire was sent only eight months before the end of the TIME study due to administrative delays, time required for ethics approval and the COVID-19 pandemic. Only 16 primary outcome events occurred subsequently among sub-study participants. We included events that occurred at any time during the TIME follow up period, but this necessarily excludes fatalities before the questionnaire was issued. It remains to be established whether our conclusions can be extended to fatal events. While there is limited published evidence of a small effect of stroke on chronotype (<1 h change in MSFsc),^43^ we are not aware of any documented effect of MI; this, combined with the relative long-term stability of chronotype,^21^ suggests that inclusion of events that occurred before questionnaire completion is unlikely to have introduced any significant bias. Another limitation caused by the relatively late implementation of the sub-study is that participants may have differed from non-sub-study participants in some unmeasured way, e.g., health consciousness, that affected their likelihood of experiencing a cardiovascular event, and a confounding interaction between this and chronotype is possible. Additionally, the questionnaire was completed after the first lockdown during the COVID-19 pandemic when various restrictions were still in place that could have affected diurnal sleep and activity patterns.^44^ Chronotype was not objectively measured using gold-standard methods (e.g. dim-light melatonin onset [DLMO], 24-hr cortisol rhythm, actigraphy) because they are laborious, expensive, and mostly rely on special conditions, however the μMCTQ has been validated against these methods.^33^ We did note that for a few participants the self-reported chronotype (MEQ question #19) did not match their μMCTQ-calculated chronotype (MSFsc) (Table S10, Appendix p 15). MSFsc should be considered the more precise measure of chronotype because it allows removal of the confounder of adaptive responses due to social schedules.^33^ Nevertheless, we confirmed a significantly reduced risk of MI regardless of whether these individuals were included in the analysis.

TIME was a highly pragmatic decentralised trial; adverse events and home BP data were self-reported and might have been subject to recall and reporting bias of unknown effect. Another limitation is that participants did not self-report the precise time within the 4-h window they were taking antihypertensives (06:00–10:00 in the morning or 20:00–00:00 in the evening), further limiting the interpretation of BP data. Variability in ingestion time may be one of many sources of statistical “noise” in the data, but we have been able to detect trends despite it. In future studies, tighter control, or better objective recording of factors like timing and dosing of drugs should result in greater precision. Morning dosing has previously been associated with better medication adherence in a non-chronotyped study population.^45^ Although the TIME study collected self-reported adherence to dosing-time, it did not collect any data on overall adherence to antihypertensive medication, we were thus unable to assess for interactions between chronotype, dosing time, and medication adherence.

Our study also has strengths. The baseline characteristics of the Chronotype sub-study cohort did not differ from those of the TIME study randomised cohort, and variables were well-balanced between groups (Table 1; Table S1 Appendix p 3). The distribution of MSFsc in the Chronotype sub-study population was well aligned with that reported in the literature.^46^ Moreover, all cardiovascular endpoints were identified using record-linked NHS hospitalisation data, and then adjudicated by an independent clinical endpoint committee blinded to the allocated dosing time and unaware of the individual chronotype. In addition, night shift workers were excluded from the TIME study, thus avoiding a potentially important confounder from the analyses.

To our knowledge, this is the first observation of a significant interaction between chronotype, a validated measure of personal circadian timing, and dosing time of usual antihypertensive medications. Our results suggest that ‘personalized circadian medicine’ in the treatment of hypertension, considering endogenous circadian rhythms of the patient (e.g., morning or evening), could provide extra protection against MI.

Our results may have direct clinical implications that can help improve outcomes in hypertensive patients. A simple chronotype questionnaire or asking patients to self-report their chronotype during a consultation, could re-direct prescription of antihypertensives to a more individual time to further reduce their MI risk.

Approximately 13.5 million people are currently living with hypertension in the UK, where each year there are 100,000 hospital admissions for MI.^47^ Thus, the potential public health and financial implications of our findings could be relevant also considering the minimal cost of this intervention.

The Chronotype sub-study results are a critical appraisal of previous studies on chronotherapy of hypertension that have not specifically stratified participants according to chronotype, missing the importance of the individual internal time when dosing antihypertensive medications. The Chronotype sub-study findings are an open call for new randomized clinical trials of chronotherapy of hypertension to confirm the importance of assessing chronotype to identify an optimal therapeutic window within the circadian cycle to maximize the cardiovascular protection of antihypertensive medications. Moreover, chronotype should be an additional factor to evaluate when designing clinical trials, and future circadian rhythm-based administration-time trials should consider individual circadian time.

In conclusion, the results of our study represent a step forward in precision medicine of hypertension, with findings that support further research aimed at tailoring therapy of hypertensive patients to individual circadian rhythms. Evening dosing of antihypertensive medications in later chronotypes, and morning dosing in earlier chronotypes were associated with a lower risk of non-fatal MI. Administration time of antihypertensive medications synchronized with a patient's chronotype might be an easy and inexpensive personalized chronotherapeutic approach to maximise the prevention of heart attacks in individuals with high BP using currently available antihypertensive drugs.

## Contributors

FP conceived the idea for the Chronotype sub-study. FP, KAD, CV, and AR participated in the design of the study. DAR programmed the study software and converted the questionnaire to an electronic version for study use. SVM did the statistical analysis. SVM and FP directly accessed and verified the underlying data reported in the manuscript. FP, KAD, and SVM wrote the first draft of the manuscript with major inputs from CV, FPC, and RM. All authors participated in the interpretation of the data and critical review of the manuscript. All authors have read and approved the final version.

## Data sharing statement

Access to de-identified participant dataset and data dictionary is available upon reasonable request to researchers who provide a methodologically sound proposal, with no prespecified restrictions on data use. Any such requests should be sent to corresponding author FP for consideration by the trial steering committee. There might be restrictions on sharing data derived by record-linkage to NHS datasets. A period of 18 months after publication of the study results should elapse before requests are made, to allow the authors to publish further analyses.

## Declaration of interests

FP, KAD, SVM, RF, DR, and RM report no conflicts of interest. CV reports to be a paid scientific consultant of the National Institutes of Mental Health, USA. CV is now an employee of IQVIA GmbH, Frankfurt am Main, Germany. AR reports travel expenses from Informa, and unpaid membership of an NIHR Trial Steering Committee. IM reports research grants from British Heart Foundation for the submitted work, and research grants from Menarini, EMA, Sanofi, HDR UK, NIHR HTA and IMI, institutional consultancy income from AstraZeneca, and personal income from AstraZeneca, Amgen, Amarin, Novartis and NovoNordisk outside the submitted work. TM reports grants from Menarini/Ipsen/Teijin, NIHR HTA and MSD outside the submitted work and from British Heart Foundation for the submitted work and personal income for consultancy from Novartis and AstraZeneca outside the submitted work. TM and IM are trustees of the Scottish Heart and Arterial Risk Prevention (SHARP) Society. FPC reports a grant from NIHR i4i outside the submitted work, consultancies from Omron Healthcare, Menarini Int., Deloitte UK, and royalties from OUP for two books. He is also unpaid advisor to the World Health Organization for work unrelated to the subject of the present study.
