# Supplementary material for: Effect of timed dosing of usual antihypertensives according to patient chronotype on cardiovascular outcomes: the Chronotype sub-study cohort of the Treatment in Morning versus Evening (TIME) study
Source: eClinicalMedicine. 2024 May 14;72:102633. doi: 10.1016/j.eclinm.2024.102633 (PMC11106533; doi:10.1016/j.eclinm.2024.102633)
Supplement: Supplementary Tables and Figures [file mmc1.pdf]

Supplementary appendix

Effect of timed dosing of usual antihypertensives according to patient chronotype on cardiovascular outcomes: the Chronotype sub-study cohort of the Treatment in Morning versus Evening (TIME) study.

Filippo Pigazzani, Kenneth A. Dyar, Steve V. Morant, Céline Vetter, Amy Rogers, Robert W.V. Flynn, David A. Rorie, Isla S. Mackenzie, Francesco P. Cappuccio, Roberto Manfredini and Thomas M. MacDonald

Table of Contents page

Supplementary Table S1: Chronotype sub-study vs TIME study baseline characteristics...3

Supplementary Table S2a: Antihypertensive use at study entry in the TIME study vs Chronotype sub-study........5

Supplementary Table S2b: Antihypertensive use in the Chronotype sub-study by dosing time group.....................5

Supplementary Table S3: Hazard ratios for non-fatal cardiovascular outcomes per hour advance in MSFsc in the intention-to-treat cohort. The interaction term tests whether the HR is different between morning and evening dosing...6

Supplementary Table S4: Hazard ratios for non-fatal cardiovascular outcomes per hour advance in MSFsc and per category later in self-reported chronotype ("definitely a morning type" = 1 to "definitely an evening type" = 4) in participants completing the study on their allocated dosing time (on-treatment analysis). The interaction term tests whether the HR is different between morning and evening dosing...7

Supplementary Table S5: Hazard ratios for non-fatal cardiovascular outcomes per hour advance in MSFsc and per category later in self-reported chronotype ("definitely a morning type"=1 to "definitely an evening type"=4) in the intention-to-treat cohort: sex differences and interactions with chronotype..8

Supplementary Table S6: Hazard ratios for self-reported adverse events per hour advance in MSFsc and per category later in self-reported chronotype ("definitely a morning type"=1 to "definitely an evening type"=4) in the intention-to-treat cohort. The interaction term tests whether the HR is different between morning and evening dosing...9

Supplementary Table S7: Adherence to allocated dosing time vs mid sleep time..11

Supplementary Table S8: Trends in home blood pressure with mid sleep time (mmHg per hour advance in MSFsc...12

Supplementary Table S9: Baseline characteristics of the Chronotype sub-study cohort by mid sleep time............13

Supplementary Table S10: MSFsc vs self-reported chronotype..15

Supplementary Figure S1: Questions asked from the Chronotype sub-study questionnaire..................................16

Supplementary Figure S2: Age and sex distribution in the Chronotype sub-study..17

Supplementary Figure S3: Distribution of mid sleep time by dosing time...18

Supplementary Figure S4: Hazard ratios for non-fatal myocardial infarction vs MSFsc (categorized as >30mins before median, within 30mins of median and >30mins after the median of 3:07AM) and vs self-reported chronotype...19

Supplementary Figure S5: Hazard ratios for non-fatal stroke vs MSFsc (categorized as >30mins before median, within 30mins of median and >30mins after the median of 3:07AM) and vs self-reported chronotype...20

Chronotype sub-study Statistical Analysis Plan..21

Supplementary Table S1: Chronotype sub-study vs TIME study baseline characteristics.

Completed chronotype questionnaire	No	Yes
	(N=15,746)	(N=5358)
Age, yrs		
Mean (SD)	65.3 (9.58)	64.4 (8.32)
Missing	160 (1.0%)	18 (0.3%)
Sex		
Female	6676 (42.4%)	2292 (42.8%)
Male	9070 (57.6%)	3066 (57.2%)
Country of residence		
Scotland	1367 (8.7%)	449 (8.4%)
England	13,820 (87.8%)	4712 (87.9%)
Wales	554 (3.5%)	196 (3.7%)
Ireland	5 (0.0%)	1 (0.0%)
Ethnicity		
Asian or Asian British	138 (0.9%)	17 (0.3%)
Black, African, Caribbean, or Black British	84 (0.5%)	14 (0.3%)
Multiple or mixed	63 (0.4%)	23 (0.4%)
Not reported	1437 (9.1%)	200 (3.7%)
Other	15 (0.1%)	12 (0.2%)
White	14,009 (89.0%)	5092 (95.0%)
Smoking status		
Never	8906 (56.6%)	3172 (59.2%)
Former	6031 (38.3%)	1976 (36.9%)
Current	699 (4.4%)	186 (3.5%)
Missing	110 (0.7%)	24 (0.4%)
Systolic blood pressure, mmHg*		
n	7314	2764
Mean (SD)	135 (13.5)	134 (12.7)
Diastolic blood pressure, mmHg*		
n	7306	2761
Mean (SD)	78.9 (9.38)	79.0 (8.97)
Body-mass index, kg/m^2 [a]		
n	14,440	5064
Mean (SD)	28.5 (4.87)	28.1 (4.78)
Cardiovascular history[b]		
Evidence of cardiovascular disease[c]	2121 (13.5%)	604 (11.3%)

Completed chronotype questionnaire	No	Yes
Previous myocardial infarction	792 (5.0%)	193 (3.6%)
Angina, requiring medical treatment	500 (3.2%)	136 (2.5%)
Previous stroke	383 (2.4%)	114 (2.1%)
Previous transient ischaemic attack	661 (4.2%)	216 (4.0%)
Peripheral vascular disease	262 (1.7%)	62 (1.2%)
Diabetes		
Yes, on medication	1610 (10.2%)	459 (8.6%)
Yes, not on medication	533 (3.4%)	164 (3.1%)
Yes, medication unknown	0 (0%)	1 (0.0%)
Asthma		
Yes, on medication	1415 (9.0%)	474 (8.8%)
Yes, not on medication	160 (1.0%)	35 (0.7%)
Kidney impairment		
Yes, on medication	90 (0.6%)	17 (0.3%)
Yes, not on medication	433 (2.8%)	142 (2.7%)
Chronic obstructive pulmonary disease		
Yes, on medication	428 (2.7%)	88 (1.6%)
Yes, not on medication	76 (0.5%)	24 (0.4%)
Arthritis, requiring medical treatment		
Yes, on medication	1115 (7.1%)	305 (5.7%)
Yes, not on medication	416 (2.6%)	162 (3.0%)
Yes, medication unknown	1 (0.0%)	0 (0%)
Number of antihypertensive medications		
Mean (SD)	1.49 (0.686)	1.50 (0.714)

Data are mean (SD) or n (%) unless otherwise stated. *Self-reported last known measurement. [a]Derived from self-reported height and weight. [b] Self-reported medical history. [c] Defined as self-reported history of angina, myocardial infarction, stroke, transient ischaemic attack, or peripheral vascular disease.

Supplementary Table S2a: Antihypertensive use at study entry in the TIME study vs Chronotype sub-study.

Completed chronotype questionnaire	Yes	No
**Antihypertensive class**		
Angiotensin-converting enzyme inhibitors	1414 (47.1%)	2297 (47.3%)
Calcium-channel blockers	1157 (38.5%)	1839 (37.9%)
Angiotensin receptor blockers	785 (26.1%)	1131 (23.3%)
Thiazide and related diuretics	591 (19.7%)	979 (20.2%)
Beta-adrenoceptor blockers	282 (9.4%)	466 (9.6%)
Alpha-adrenoceptor blockers	153 (5.1%)	258 (5.3%)
Other diuretics	102 (3.4%)	158 (3.3%)
Other	49 (1.6%)	106 (2.2%)

Supplementary Table S2b: Antihypertensive use in the Chronotype sub-study by dosing time group.

Antihypertensive class	Evening dosing	Morning dosing
Angiotensin-converting enzyme inhibitors	668 (45.8%)	746 (48.2%)
Calcium-channel blockers	551 (37.8%)	606 (39.2%)
Angiotensin receptor blockers	406 (27.9%)	379 (24.5%)
Thiazide and related diuretics	260 (17.8%)	331 (21.4%)
Beta-adrenoceptor blockers	139 (9.5%)	143 (9.2%)
Alpha-adrenoceptor blockers	73 (5.0%)	80 (5.2%)
Other diuretics	58 (4.0%)	44 (2.8%)
Other	24 (1.6%)	25 (1.6%)

Supplementary Table S3: Hazard ratios for non-fatal cardiovascular outcomes per hour advance in MSFsc and per category later in self-reported chronotype ("definitely a morning type" = 1 to "definitely an evening type" = 4) in the intention-to-treat cohort· The interaction term tests whether the HR is different between morning and evening dosing.

			Unadjusted event rate			Linear trend	
**Outcome**	**Chronotype variable**	**Dosing time**	**Patient years**	**Events**	**Rate[a]**	**Hazard ratio**	**Interaction**
MI or stroke	Mid sleep time	Morning	14,589	54	3.7	1.46 (1.14,1.86)	p=0.036
		Evening	13,532	46	3.4	0.96 (0.70,1.30)	
	Self-reported chronotype	Morning	14,589	54	3.7	1.32 (1.02,1.72)	p=0.032
		Evening	13,532	46	3.4	0.86 (0.63,1.16)	
MI	Mid sleep time	Morning	14,637	30	2.0	1.62 (1.18,2.22)	p<0.001
		Evening	13,588	26	1.9	0.66 (0.44,1.00)	
	Self-reported chronotype	Morning	14,637	30	2.0	1.27 (0.89,1.80)	p=0.006
		Evening	13,588	26	1.9	0.58 (0.36,0.91)	
Stroke	Mid sleep time	Morning	14,672	23	1.6	1.44 (1.02,2.03)	p=0.726
		Evening	13,608	19	1.4	1.59 (1.02,2.49)	
	Self-reported chronotype	Morning	14,672	23	1.6	1.31 (0.88,1.96)	p=0.742
		Evening	13,608	19	1.4	1.19 (0.77,1.83)	
Heart failure	Mid sleep time	Morning	14,713	5	0.3	1.11 (0.40,3.06)	p=0.997
		Evening	13,657	6	0.4	1.11 (0.42,2.92)	
	Self-reported chronotype	Morning	14,713	5	0.3	1.18 (0.50,2.77)	p=0.660
		Evening	13,657	6	0.4	1.53 (0.70,3.35)	

[a] Events per thousand patient years

Supplementary Table S4: Hazard ratios for non-fatal cardiovascular outcomes per hour advance in MSFsc and per category later in self-reported chronotype ("definitely a morning type" = 1 to "definitely an evening type" = 4) in participants completing the study on their allocated dosing time (on-treatment analysis). The interaction term tests whether the HR is different between morning and evening dosing.

			Unadjusted event rate			Linear trend	
**Outcome**	**Chronotype variable**	**Dosing time**	**Patient years**	**Events**	**Rate[a]**	**Hazard ratio**	**Interaction**
MI or stroke	Mid sleep time	Morning	13,353	49	3.7	1.40 (1.07,1.82)	p=0.108
		Evening	10,952	36	3.3	0.98 (0.69,1.38)	
	Self-reported chronotype	Morning	13,353	49	3.7	1.26 (0.95,1.66)	p=0.149
		Evening	10,952	36	3.3	0.92 (0.66,1.28)	
MI	Mid sleep time	Morning	13,403	29	2.2	1.68 (1.23,2.30)	p=0.002
		Evening	10,982	20	1.8	0.70 (0.44,1.11)	
	Self-reported chronotype	Morning	13,403	29	2.2	1.27 (0.89,1.82)	p=0.016
		Evening	10,982	20	1.8	0.60 (0.36,1.01)	
Stroke	Mid sleep time	Morning	13,425	20	1.5	1.35 (0.90,2.02)	p=0.671
		Evening	10,981	15	1.4	1.55 (0.93,2.57)	
	Self-reported chronotype	Morning	13,425	20	1.5	1.19 (0.77,1.83)	p=0.865
		Evening	10,981	15	1.4	1.25 (0.78,2.03)	
Heart failure	Mid sleep time	Morning	13,473	5	0.4	1.07 (0.39,2.92)	p=0.535
		Evening	11,002	4	0.4	0.65 (0.19,2.20)	
	Self-reported chronotype	Morning	13,473	5	0.4	1.19 (0.50,2.84)	p=0.469
		Evening	11,002	4	0.4	1.95 (0.69,5.49)	

[a] Events per thousand patient years

Supplementary Table S5: Hazard ratios for non-fatal cardiovascular outcomes per hour advance in MSFsc and per category later in self-reported chronotype ("definitely a morning type" = 1 to "definitely an evening type" = 4) in the intention-to-treat cohort: sex differences and interactions with chronotype.

							Hazard ratio (95% confidence interval)		p-value for
**Outcome**	**Chronotype variable**	**Dosing time**	**Sex**	**Patient years**	**Events**	**Rate**	**Male vs female**	**Chronotype[1]**	**interaction**
Primary outcome	Mid sleep time	Morning	Male	8305	38	4.6	1.85 (0.98,3.49)	1.49 (1.16,1.92)	0.314
			Female	6284	16	2.5			
		Evening	Male	7701	38	4.9	2.90 (1.33,6.34)	0.96 (0.71,1.30)	0.710
			Female	5831	8	1.4			
	Self-reported chronotype	Morning	Male	8305	38	4.6	1.87 (0.99,3.52)	1.28 (1.00,1.65)	0.528
			Female	6284	16	2.5			
		Evening	Male	7701	38	4.9	2.88 (1.32,6.28)	0.90 (0.70,1.17)	0.914
			Female	5831	8	1.4			
MI	Mid sleep time	Morning	Male	8325	25	3.0	3.82 (1.42,10.27)	1.66 (1.19,2.32)	0.067
			Female	6312	5	0.8			
		Evening	Male	7743	21	2.7	2.40 (0.88,6.52)	0.67 (0.45,0.99)	0.409
			Female	5846	5	0.9			
	Self-reported chronotype	Morning	Male	8325	25	3.0	3.86 (1.44,10.33)	1.25 (0.90,1.73)	0.131
			Female	6312	5	0.8			
		Evening	Male	7743	21	2.7	2.41 (0.89,6.53)	0.67 (0.46,0.97)	0.507
			Female	5846	5	0.9			
Stroke	Mid sleep time	Morning	Male	8376	13	1.6	0.82 (0.34,1.99)	1.48 (1.03,2.12)	0.889
			Female	6296	10	1.6			
		Evening	Male	7757	16	2.1	3.80 (1.07,13.48)	1.58 (1.01,2.48)	0.129
			Female	5851	3	0.5			
	Self-reported chronotype	Morning	Male	8376	13	1.6	0.83 (0.34,2.00)	1.31 (0.88,1.94)	0.576
			Female	6296	10	1.6			
		Evening	Male	7757	16	2.1	3.76 (1.07,13.22)	1.35 (0.89,2.03)	0.489
			Female	5851	3	0.5			

[1] Per hour for mid sleep time and per category for self-reported chronotype

Supplementary Table S6: Hazard ratios for self-reported adverse events per hour advance in MSFsc and per category later in self-reported chronotype ("definitely a morning type" = 1 to "definitely an evening type" = 4) in the intention-to-treat cohort. The interaction term tests whether the HR is different between morning and evening dosing.

			Unadjusted event rate			Linear trend with chronotype variable	
**Outcome**	**Chronotype variable**	**Dosing time**	**Patient years**	**Events**	**Rate**	**Hazard ratio (95% confidence interval)**	**Interaction**
Dizziness	Mid sleep time	Morning	11,090	1341	120.9	1.00 (0.95,1.06)	p=0.526
		Evening	10,407	1157	111.2	1.03 (0.97,1.09)	
	Self-reported chronotype	Morning	11,090	1341	120.9	1.05 (0.99,1.10)	p=0.633
		Evening	10,407	1157	111.2	1.03 (0.97,1.09)	
Falls	Mid sleep time	Morning	13,598	522	38.4	1.12 (1.02,1.22)	p=0.153
		Evening	12,735	428	33.6	1.01 (0.92,1.12)	
	Self-reported chronotype	Morning	13,598	522	38.4	1.03 (0.94,1.12)	p=0.903
		Evening	12,735	428	33.6	1.02 (0.93,1.12)	
Visits to toilet	Mid sleep time	Morning	11,434	1164	101.8	1·00 (0.94,1.06)	p=0.562
		Evening	9,895	1208	122.1	1.02 (0.96,1.09)	
	Self-reported chronotype	Morning	11,434	1164	101.8	1.02 (0.96,1.08)	p=0.707
		Evening	9,895	1208	122.1	1.03 (0.98,1.10)	
Sleep disturbance	Mid sleep time	Morning	10,819	1372	126.8	0.94 (0.89,1.00)	p=0.631
		Evening	9,531	1339	140.5	0.92 (0.87,0.98)	
	Self-reported chronotype	Morning	10,819	1372	126.8	1.02 (0.97,1.07)	p=0.315
		Evening	9,531	1339	140.5	0.98 (0.93,1.03)	
Indigestion	Mid sleep time	Morning	11,954	1060	88.7	1.10 (1.03,1.17)	p=0.447
		Evening	11,219	925	82.5	1.06 (0.99,1.14)	
	Self-reported chronotype	Morning	11,954	1060	88.7	1.06 (1.00,1.12)	p=0.860
		Evening	11,219	925	82.5	1.07 (1.00,1.14)	
Diarrhea	Mid sleep time	Morning	12,797	777	60.7	1.09 (1.01,1.17)	p=0.320
		Evening	12,113	639	52.8	1.03 (0.95,1.12)	
	Self-reported chronotype	Morning	12,797	777	60.7	1.10 (1.03,1.18)	p=0.683
		Evening	12,113	639	52.8	1.08 (1.00,1.16)	
Generally unwell	Mid sleep time	Morning	11,900	1060	89.1	1.07 (1.01,1.14)	p=0.756
		Evening	10,902	977	89.6	1.06 (0.99,1.13)	
	Self-reported chronotype	Morning	11,900	1060	89.1	1.10 (1.04,1.17)	p=0.791
		Evening	10,902	977	89.6	1.09 (1.02,1.16)	
Muscle aches	Mid sleep time	Morning	10,734	1412	131.5	1.03 (0.98,1.09)	p=0.536
		Evening	10,021	1275	127.2	1.06 (1.00,1.12)	

			Unadjusted event rate			Linear trend with chronotype variable	
**Outcome**	**Chronotype variable**	**Dosing time**	**Patient years**	**Events**	**Rate**	**Hazard ratio (95% confidence interval)**	**Interaction**
	Self-reported chronotype	Morning	10,734	1412	131.5	1.03 (0.98,1.09)	p=0.990
		Evening	10,021	1275	127.2	1.03 (0.98,1.09)	
Other	Mid sleep time	Morning	24,028	1986	82.7	1.09 (0.99,1.19)	p=0.183
		Evening	21,787	1890	86.7	0.99 (0.90,1.09)	
	Self-reported chronotype	Morning	24,028	1986	82.7	1.15 (1.05,1.25)	p=0.026
		Evening	21,787	1890	86.7	0.99 (0.90,1.09)	

Supplementary Table S7: Adherence to allocated dosing time vs mid sleep time.

	Morning dosing		Evening dosing	
**Mid sleep time**	n	% switched	n	% switched
>30mins before median	662	5.6	673	16.8
Within 30mins of median	1272	6.4	1170	18.4
>30mins after median	750	8.1	663	17.6
p-value (logistic regression)	0.144		0.647	

Supplementary Table S8: Trends in home blood pressure with mid sleep time (mmHg per hour advance in MSFsc).

			Unadjusted		Adjusted[a]	
**Blood pressure**		Dosing time		p value		p value
**Diastolic**	AM	Evening	0.06 (-0.41,0.52)	0.810	0.05 (-0.39,0.48)	0.824
		Morning	-0.13 (-0.58,0.33)	0.583	-0.10 (-0.53,0.33)	0.646
	PM	Evening	0.30 (-0.15,0.75)	0.189	0.32 (-0.11,0.74)	0.147
		Morning	0.01 (-0.43,0.45)	0.960	0.05 (-0.36,0.47)	0.800
**Systolic**	AM	Evening	0.55 (-0.07,1.17)	0.084	0.49 (-0.12,1.10)	0.114
		Morning	0.05 (-0.56,0.66)	0.875	0.01 (-0.58,0.61)	0.965
	PM	Evening	0.92 (0.29,1.56)	0.004	0.87 (0.25,1.49)	0.006
		Morning	0.29 (-0.34,0.91)	0.366	0.26 (-0.35,0.87)	0.407

[a] Other covariates included in the model were: gender, age, smoking status, history of heart attack, history of stroke, number of antihypertensive drugs and their half-life.

Supplementary Table S9: Baseline characteristics of the Chronotype sub-study cohort by mid sleep time.

**Mid sleep time (MSFsc)**	**>30mins before median** (n = 1335)	**Within 30mins of median** (n = 2442)	**>30mins after median** (n = 1414)
Age, yrs			
Mean (SD)	64.0 (8.29) [1333]	64.7 (8.14) [2431]	64.0 (8.50) [1412]
Missing	2 (0.1%)	11 (0.5%)	2 (0.1%)
Gender			
Female	549 (41.1%)	1018 (41.7%)	674 (47.7%)
Male	786 (58.9%)	1424 (58.3%)	740 (52.3%)
Place of residence			
Scotland	90 (6.7%)	197 (8.1%)	147 (10.4%)
England	1197 (89.7%)	2152 (88.1%)	1224 (86.6%)
Wales	48 (3.6%)	92 (3.8%)	43 (3.0%)
Ireland	0 (0%)	1 (0.0%)	0 (0%)
Ethnicity			
Asian or Asian British	1 (0.1%)	5 (0.2%)	11 (0.8%)
Black, African, Caribbean, or Black British	2 (0.1%)	8 (0.3%)	3 (0.2%)
Multiple or mixed	6 (0.4%)	10 (0.4%)	6 (0.4%)
Not reported	53 (4.0%)	83 (3.4%)	58 (4.1%)
Other	4 (0.3%)	5 (0.2%)	2 (0.1%)
White	1269 (95.1%)	2331 (95.5%)	1334 (94.3%)
Smoking status			
Never	792 (59.3%)	1493 (61.1%)	807 (57.1%)
Former	491 (36.8%)	875 (35.8%)	530 (37.5%)
Current	46 (3.4%)	63 (2.6%)	70 (5.0%)
Missing	6 (0.4%)	11 (0.5%)	7 (0.5%)
Systolic blood pressure*			
Mean (SD)	134 (13.1) [681]	134 (12.4) [1,287]	134 (12.7) [716]
Diastolic blood pressure*			
Mean (SD)	79.3 (8.97) [681]	78.7 (8.77) [1286]	79.2 (9.19) [715]
Total cholesterol			
Mean (SD)	4.82 (1.13) [366]	4.79 (1.16) [681]	4.78 (1.27) [406]
Body-mass index, kg/m^2 [a]			
Mean (SD)	27.9 (4.79) [1261]	27.7 (4.52) [2324]	29.0 (5.10) [1327]

Mid sleep time (MSFsc)	**>30mins before median** (n = 1335)	**Within 30mins of median** (n = 2442)	**>30mins after median** (n = 1414)
Evidence of cardiovascular disease[c]			
Yes	136 (10.2%)	259 (10.6%)	182 (12.9%)
Previous myocardial infarction			
Yes	35 (2.6%)	91 (3.7%)	53 (3.7%)
Angina, requiring medical treatment			
Yes, on medication	34 (2.5%)	55 (2.3%)	43 (3.0%)
Yes, not on medication	11 (0.8%)	8 (0.3%)	9 (0.6%)
Previous stroke			
Yes	24 (1.8%)	43 (1.8%)	37 (2.6%)
Previous transient ischaemic attack			
Yes	49 (3.7%)	98 (4.0%)	61 (4.3%)
Peripheral vascular disease			
Yes, medication unknown	8 (0.6%)	26 (1.1%)	24 (1.7%)
Diabetes			
Yes, on medication	103 (7.7%)	158 (6.5%)	177 (12.5%)
Yes, not on medication	42 (3.1%)	78 (3.2%)	40 (2.8%)
Yes, medication unknown	0 (0%)	0 (0%)	1 (0.1%)
Asthma			
Yes, on medication	106 (7.9%)	202 (8.3%)	156 (11.0%)
Yes, not on medication	8 (0.6%)	18 (0.7%)	7 (0.5%)
Kidney impairment			
Yes, on medication	3 (0.2%)	6 (0.2%)	7 (0.5%)
Yes, not on medication	32 (2.4%)	59 (2.4%)	44 (3.1%)
Chronic obstructive pulmonary disease			
Yes, on medication	22 (1.6%)	34 (1.4%)	24 (1.7%)
Yes, not on medication	4 (0.3%)	9 (0.4%)	9 (0.6%)
Arthritis, requiring medical treatment			
Yes, on medication	75 (5.6%)	119 (4.9%)	101 (7.1%)
Yes, not on medication	34 (2.5%)	68 (2.8%)	49 (3.5%)
Number of antihypertensive medications			
Mean (SD)	1.49 (0.699) [739]	1.48 (0.711) [1370]	1.54 (0.725) [810]

Data are mean (SD) or n (%) unless otherwise stated. *Self-reported last known measurement. [a]Derived from self-reported height and weight. [b] Self-reported medical history. [c] Defined as self-reported history of angina, myocardial infarction, stroke, transient ischaemic attack, or peripheral vascular disease.

Supplementary Table S10: MSFsc vs self-reported chronotype.

**Chronotype**	**Mid sleep time (MSFsc, median 3:07 AM)**			
	**>30mins before median**	**Within 30mins of median**	**>30mins after median**	Total
**Definitely a morning type**	792 (15.3%)	701 (13.5%)	125 (2.4%)	1618 (31.2%)
**More morning than evening**	424 (8.2%)	1078 (20.8%)	310 (6.0%)	1812 (34.9%)
**More evening than morning**	100 (1.9%)	528 (10.2%)	525 (10.1%)	1153 (22.2%)
**Definitely an evening type**	19 (0.4%)	135 (2.6%)	454 (8.7%)	608 (11.7%)
Total	1335 (25.7%)	2442 (47.0%)	1414 (27.2%)	5191 (100.0%)

Supplementary Figure S1: Questions asked from the Chronotype sub-study questionnaire.

Part 1: Micro Munich ChronoType Questionnaire (μMCTQ) + MEQ #19:

The following section will ask you questions regarding your typical sleep and wake times on work- and work-free days. Please estimate an average of your 'normal' sleep times over the past 6 weeks when you were able to follow your usual routines.

1. **Normally, I work _____ days per week.**

Please answer all the following questions even if you do not work or work 7 days per week. Please don't forget to mark AM or PM.

2. **On WORKDAYS …**
   I normally fall asleep at ____:____ ☐ AM ☐ PM (note, this is NOT when you physically get into bed)
   I normally wake up at ____:____ ☐ AM ☐ PM (note, this is NOT when you physically get out of bed)

3. **On WORK-FREE DAYS when I DON'T use an alarm clock …**
   I normally fall asleep at ____:____ ☐ AM ☐ PM (note, this is NOT when you physically get into bed)
   I normally wake up at ____:____ ☐ AM ☐ PM (note, this is NOT when you physically get out of bed)

4. **One hears about "morning types" and "evening types." Which one of these types do you consider yourself to be?**
   ☐ Definitely a morning type
   ☐ Rather more a morning type than an evening type
   ☐ Rather more an evening type than a morning type
   ☐ Definitely an evening type

Supplementary Figure S2: Age and sex distribution in the Chronotype sub-study.

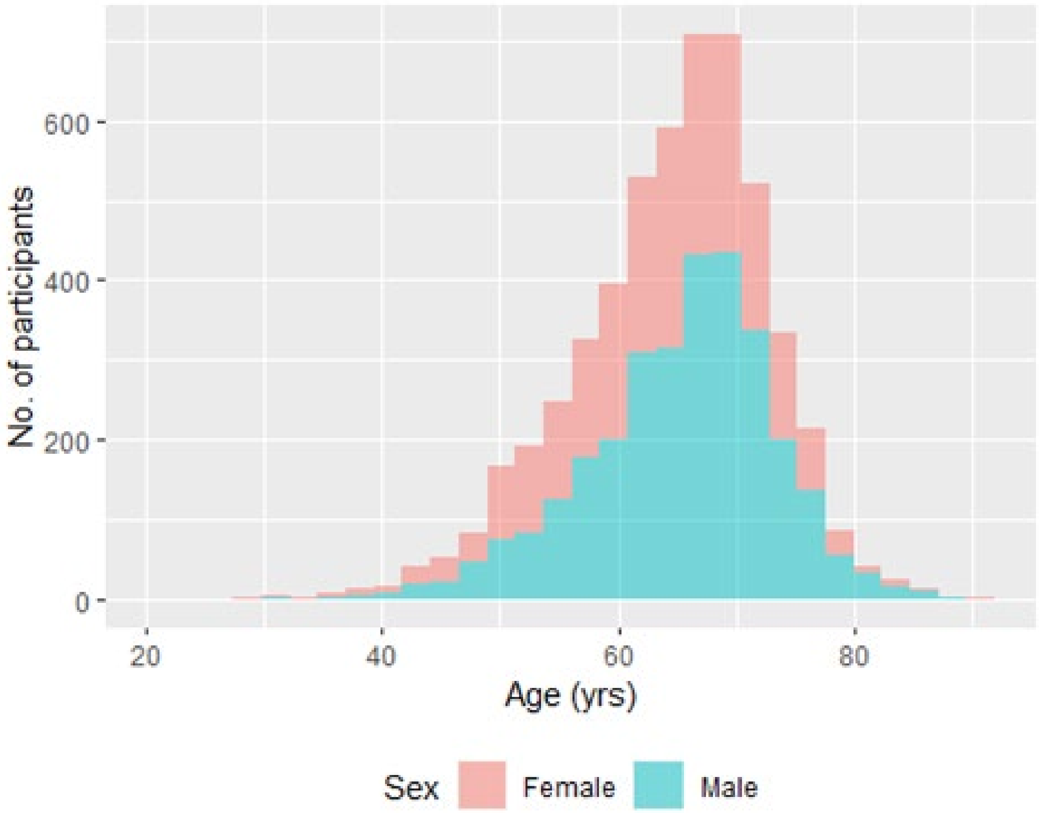

Supplementary Figure S3: Distribution of mid sleep time by dosing time.

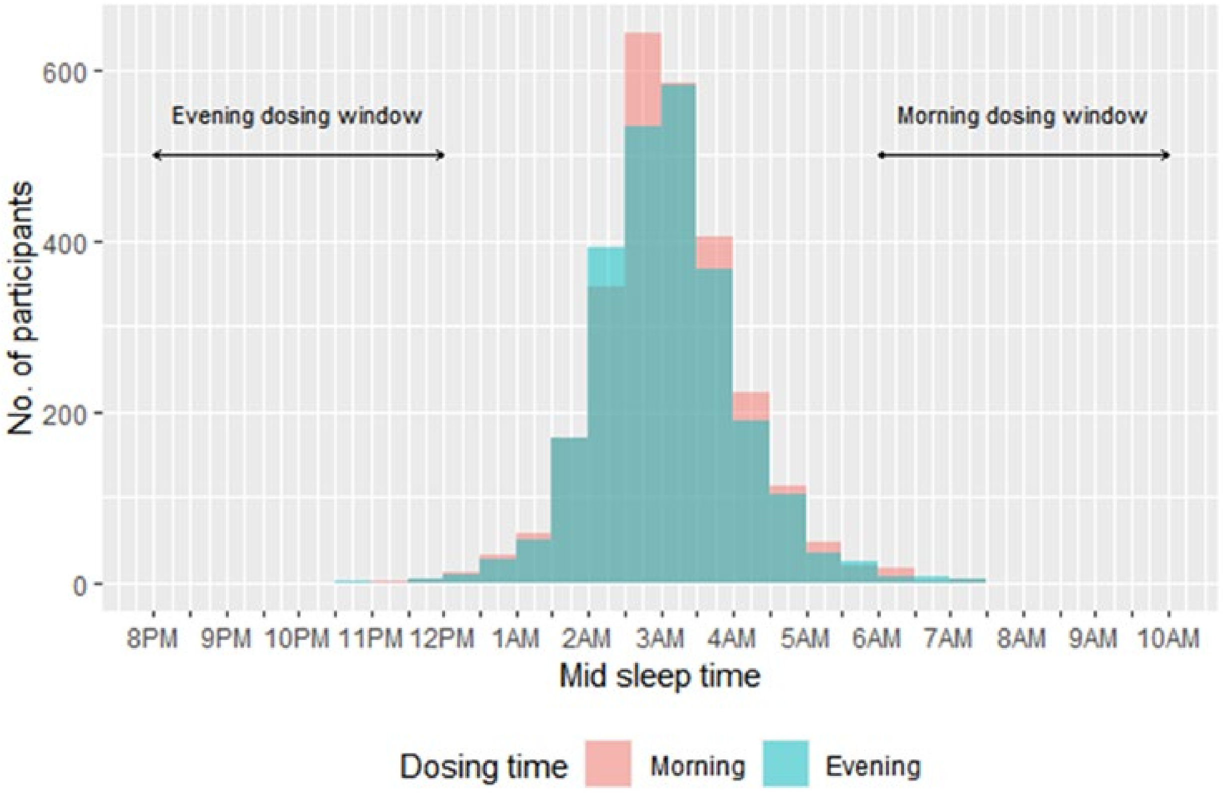

Supplementary Figure S4: Hazard ratios for non-fatal myocardial infarction vs MSFsc (categorized as >30mins before median, within 30mins of median and >30mins after the median of 3:07AM) and vs self-reported chronotype.

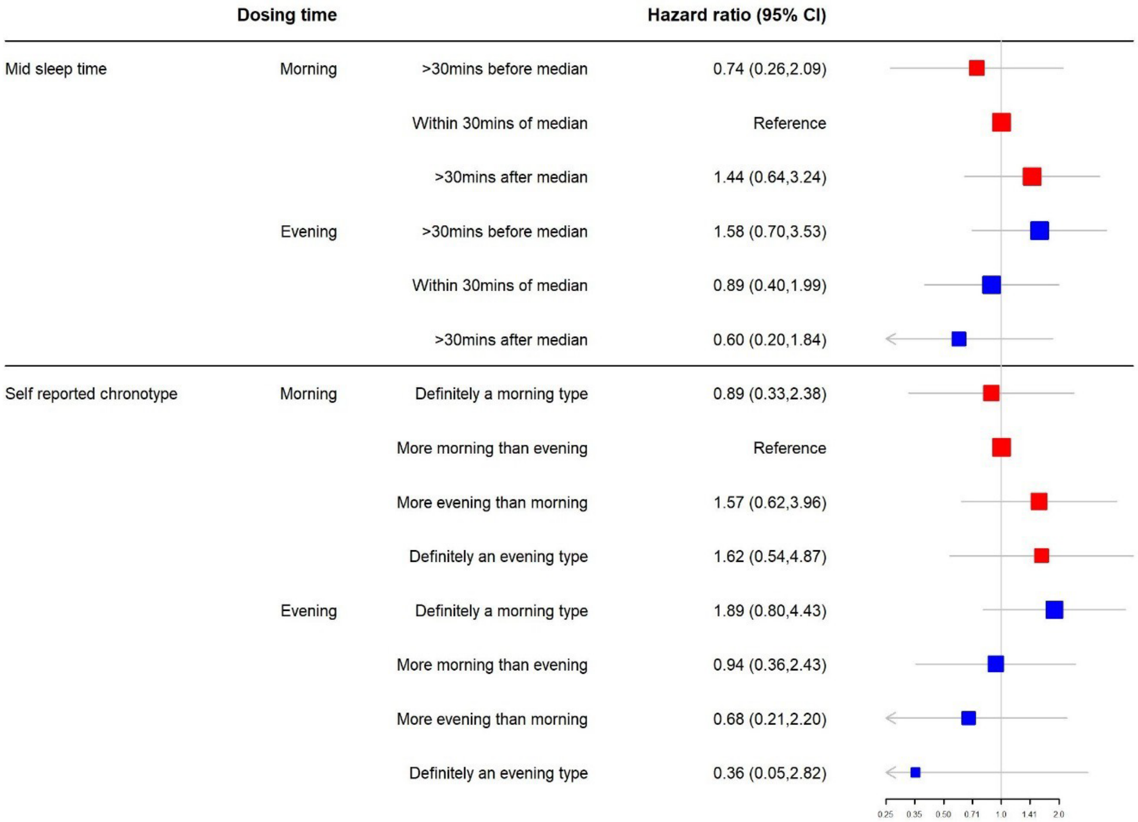

Supplementary Figure S5: Hazard ratios for non-fatal stroke vs MSFsc (categorized as >30mins before median, within 30mins of median and >30mins after the median of 3:07AM) and vs self-reported chronotype.

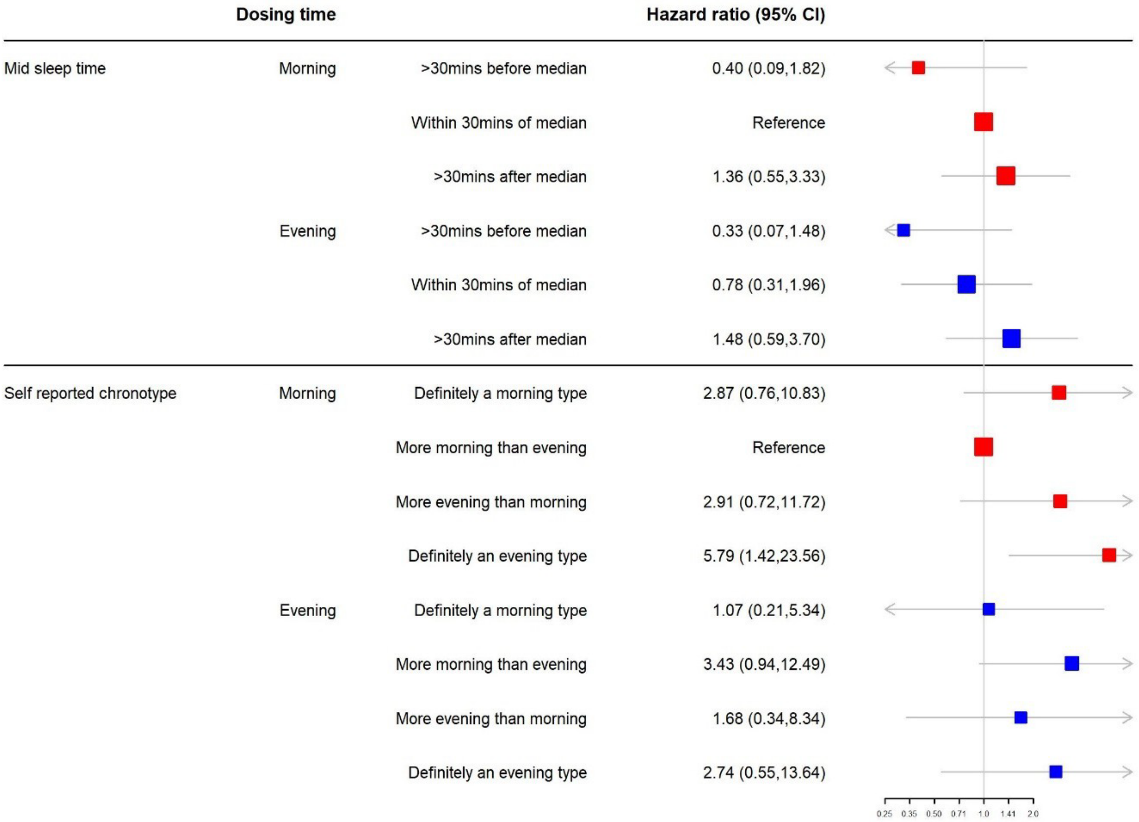

TIME CHRONOTYPE SUB-STUDY: STATISTICAL ANALYSIS PLAN

Study Title	Treatment in Morning versus Evening Study (TIME): Chronotype Sub-study
Funder	British Heart Foundation (BHF)
Sponsor	University of Dundee
Protocol Version	12.0 (10-02-21)
SAP Version	1.0 (17-03-23)
Prepared by	Dr Steve Morant, Study Statistician
Signature/Date	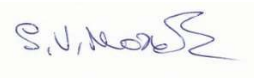 26/3/23
Approved by	Dr Steve Morant, Study Statistician
Signature/Date	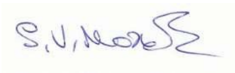 26/3/23
Approved by	Professor Tom MacDonald, TIME Study Chief Investigator
Signature/Date	

CONTENTS

1.Aim 23
2.SUB-STUDY Background 23
2.1. Circadian Rhythm and Social Jetlag 23
2.2. How to measure the biological clock? 24
3.Research questions 25
4.Methods· 26
4.1. Participants 26
4.2. Determination of chronotypes and social jetlag 26
4.3. Physical activity 26
5.Analysis plan 26
5.1. GENERAL PRINCIPLES 26
5.2. STUDY PROTOCOL 27
5.3. SOFTWARE 27
5.4. CLINICAL OUTCOMES 27
5.5. BASELINE CHARACTERISTICS 28
6.REFERENCES 30

1. AIM

This sub-study of the TIME study aims to establish if there is a link between the pattern of circadian rhythm of the trial participants with TIME study cardiovascular endpoints, adverse events and adherence to treatment

2. SUB-STUDY BACKGROUND

The TIME study aims to determine if evening dosing of blood pressure lowering medication is more effective at preventing ischaemic heart disease, strokes and cardiovascular deaths than morning dosing.

The background to the main TIME trial is detailed in the main study protocol

[1] and is not repeated here.

2.1. CIRCADIAN RHYTHM AND SOCIAL JETLAG

Living organisms have adapted their biological rhythm to the different phases of the day that are an expression of the Earth's axial rotation which causes day/night cycles around 24 hours. An internal biological clock predates day and night cycles and facilitates the balance of physiological activities (e·g· blood pressure, sleep patterns, body temperature, hormone release, blood glucose, metabolism and behaviour). This inherent physiological system is known as circadian rhythm.[2]

Circadian components have been found in gene expression of a significant number of genes which code for drug targets.[3,4,5,6,7] A relationship between medication dosing time and their effectiveness has been demonstrated in humans.[8] The dosing time seems potentially critical to managing disease, such as hypertension, considering the short half-life of several medications used in daily practice.

Moreover, emerging evidence suggests that a chronic misalignment between our internal clock and the external environment/social time, defined as Social Jetlag (SJL), contributes to the development of pathological states, including metabolic disorders and hypertension.[9,10,11]

These findings raise the question of whether giving antihypertensives according to body rhythms can improve their therapeutic effects and safety profile and can also help in preventing the potential negative outcomes of a circadian disruption.

2.2. HOW TO MEASURE THE BIOLOGICAL CLOCK?

Human beings usually show individual phase preference for activity and sleep, which allow one to be classified as a distinct chronotype. Morning chronotypes, or “Larks”, rise early and show peak alertness in the mid-morning hours. Evening chronotypes, or “Owls” tend to be late-risers and exhibit peak alertness later in the day, often late into the evening.

The onset of melatonin secretion under dim light conditions (the Dim Light Melatonin Onset or DLMO) is the single most accurate marker for assessing the circadian pacemaker· However, this measurement is expensive and burdensome.

Over the last few decades, the assessment of chronotypes has been performed using different kinds of questionnaires [Morningness-Eveningness Questionnaire (MEQ) [12] ; Munich ChronoType Questionnaire (MCTQ) [13] and recently an ultra-short version of the Munich ChronoType Questionnaire (µMCTQ)[14]] designed to measure self-reported preferences for sleep and lifestyle patterns, including timing of peak activity and alertness during the day, and timing of the sleep-wake cycle.

Among these questionnaires, the MCTQ uses the midpoint between sleep on- and offset on free days (midsleep on free days, MSF) to assess chronotype· The midpoint of sleep has been found to be one of the most accurate behavioural markers for circadian phase.[15]

The MCTQ is the only questionnaire which considers workdays and work-free days separately allowing researchers to quantify the Social Jetlag (the chronic discrepancy between a person's biological clock and the social clock) for an individual.[16]

If medication effects can be enhanced or attenuated based on the interaction between patient chronotype and the timing of medicine administration, it might be possible to see this effect in the TIME population.

3. RESEARCH QUESTIONS

This sub-study will address the following research questions:

1. Does matching chronotype to timing of administration of antihypertensives result in improved cardiovascular outcomes?
2. Does matching chronotype to timing of administration of antihypertensives result in altered adverse event rates?
3. Does matching chronotype to timing of administration of antihypertensives result in improved adherence to the hypertensive treatment?
4. Does matching chronotype to timing of administration of antihypertensives result in improved blood pressure control?
5. Are any of these effects dependent on the half-life of the antihypertensive drug?
6. Does the Social Jetlag affect cardiovascular outcomes?
7. Can physical activity mitigate risks associated with Social Jetlag?
8. Does matching chronotype to timing of administration mitigate risks associated with Social Jetlag?

4. METHODS

4.1. PARTICIPANTS

Patients participating in the TIME Study were invited to participate in the chronotype sub- study via email. If they consented to taking part using an online patient information sheet and consent form, they were asked to complete an online questionnaire about their sleeping and physical activity pattern.

4.2. DETERMINATION OF CHRONOTYPES AND SOCIAL JETLAG

The µMCTQ will be used to assign participants into different chronotype groups and to measure their social jetlag.[16] The question 19th of the MEQ will give an alternative assessment, in particular, in participants with sleep disorders. This is in keeping with the UK biobank questionnaire.

4.3. PHYSICAL ACTIVITY

Using the general practice physical activity questionnaire (GPPAQ) [17] will be identify patients who are inactive, moderately inactive, active or moderately active. At active patients will be asked at what time of the day they usually perform exercise: morning (6:00-12:00); afternoon (12:00-18:00), evening (18:00-24:00) or night (0:00-6:00).

5. ANALYSIS PLAN

5.1. GENERAL PRINCIPLES

This study is exploratory and is intended to identify hypothesis that can be tested by further research. The analyses will use an as-treated approach and the emphasis will be on parameter estimation rather than hypothesis testing.

Effects of drug half life will be explored using the entire TIME cohort.

The chronotype questionnaire was sent out on 3rd August 2020, near the end of the TIME study follow up period. Participants who had died before this date could not have completed it and participants with non-fatal primary outcome events are under-represented in the sub-study population: it includes 25% of the TIME study participants but only 13% of the primary outcome events. Redefining the origin for time-to-event analyses as 3rd August 2020 in the sub-study cohort would eliminate this immortal time bias, but only 16 primary outcome events occurred after this date. Therefore, we propose to analyse the chronotype sub-study using the TIME study time origin, accepting that absolute hazards will be biased but relying on the assumption that hazard ratios will not.

5.2. STUDY PROTOCOL

The current TIME Chronotype sub-study protocol at the time of writing is Version 4, dated 05th November 2019. A brief description of the TIME Chronotype sub-study can be found on the TIME study protocol Version 12.0, dated 10th February 2021, published online in October 2022.[18]

5.3. SOFTWARE

Analyses will be carried out using R for Windows v4.1.1.

5.4. CLINICAL OUTCOMES

The primary outcome in the TIME study was the time from randomisation to first event for the composite of vascular death, hospitalisation for non-fatal MI or non-fatal stroke.

The primary outcome in the TIME study is also an outcome in the Chronotype sub-study and it will be analysed using Cox proportional hazards models if the proportional hazards assumptions are met.

Similar models will be used to analyses time to non-fatal secondary endpoints (hospitalisation for MI, stroke or heart failure). Logistic regression models will be used to analyse the occurrence of nine pre-specified adverse events (dizziness/light headedness, falls, excessive visits to the toilet, sleep problems, upset stomach/indigestion, diarrhoea, feeling generally less well, muscle aches, others).

The models will include: evening vs morning dosing (last known dosing time), adherence (fully adherent, switched dosing time but last know time same as the allocated time, switched dosing time and last known time different from the allocated time), mid-sleep time, social jetlag, self-reported chronotype and half-life of antihypertensives. A term for the interaction between time of administration and chronotype will estimate the effect of discordant treatment (early chronotypes taking their study drug in the evening and late chronotypes taking it in the morning) *vs* concordant treatment, or subgroup analyses will be carried out. If social jetlag proves to be predictive of cardiovascular outcomes, a measure of physical activity will also be included to estimate any mitigating effect it might [illegible]

5.5. BASELINE CHARACTERISTICS

The models will also include any of the following baseline covariates specified in the TIME trial protocol that are predictive of the outcome:

- Demographics
    - Age
    - Sex
- Cardiovascular Risk Factors
    - Family History of cardiovascular disease
    - Systolic blood pressure

  - Diastolic blood pressure
  - Total cholesterol
  - BMI
  - Smoking status

- Medical History
  - Prior heart attack
  - Prior stroke
  - Impaired kidney function
  - Peripheral vascular disease
  - Any CVD (MI, CVA, PVD)
  - Diabetes
  - COPD
  - Chronic arthritis

- Medication use
  - Number of antihypertensive medications
  - Diuretic
  - ACE inhibitor
  - ARB
  - CCB
  - Beta blocker
  - Alpha-blocker
  - Other antihypertensive medication

We will summarise differences in these baseline characteristics between chronotypes.

We will assess the agreement between the two methods (either mid-sleep time or self-reported classification) used to assess the chronotype.

6. REFERENCES

[1] Rorie DA, Rogers A, Mackenzie IS, et al· Methods of a large prospective, randomised, open-label, blinded end-point study comparing morning versus evening dosing in hypertensive patients: the Treatment In Morning versus Evening (TIME) study. BMJ Open. 2016 Feb;6(2):e010313. DOI: 10.1136/bmjopen-2015-010313. PMID: 26861939; PMCID: PMC4762112.

[2] Partch CL, Green CB, Takahashi JS. Molecular architecture of the mammalian circadian clock. Trends Cell Biol. 2014 Feb;24(2):90-9. Review.

[3] Zhang R, Lahens NF, Ballance HI, Hughes ME, Hogenesch JB. A circadian gene expression atlas in mammals: Implications for biology and medicine. Proc Natl Acad Sci U S A. 2014;111(45):16219–24.

[4] Barclay NL, Eley TC, Buysse DJ, Archer SN, Gregory AM. Diurnal preference and sleep quality: same genes? A study of young adult twins. Chronobiol Int (2010) 27:278–96.

[5] Archer SN, Robilliard DL, Skene DJ, Smits M, Williams A, Arendt J, et al. A length polymorphism in the circadian clock gene Per3 is linked to delayed sleep phase syndrome and extreme diurnal preference. Sleep (2003) 26:413–5.

[6] Archer, S. N., Viola, A. U., Kyriakopoulou, V., von Schantz, M. & Dijk, D. Inter-individual differences in habitual sleep timing and entrained phase of endogenous circadian rhythms of BMAL1, PER2 and PER3 mRNA in human leukocytes. Sleep 31, 608–617 (2008).

[7] Ferrante, A. et al. Diurnal Preference Predicts Phase Differences in Expression of Human Peripheral Circadian Clock Genes. J. Circadian Rhythms 13, 1–7 (2015).

[8] Ruben MD, Smith DF, FitzGerald GA, Hogenesch JB. Dosing time matters. Science.2019 Aug 9;365(6453):547-549.

[9] Roenneberg T, Merrow M. The Circadian Clock and Human Health. Curr Biol. 2016 May 23;26(10):R432-43. doi: 10.1016/j.cub.2016.04.011. Review.

[10] Ohlander J, Keskin MC, Stork J, Radon K. Shift work and hypertension: Prevalence and analysis of disease pathways in a German car manufacturing company. Am J Ind Med. 2015 May;58(5):549-60

[11] Reutrakul S, Knutson KL. Consequences of Circadian Disruption on Cardiometabolic Health. Sleep Med Clin. 2015 Dec;10(4):455-68. Review.

[12] Horne, J. A. & Östberg, O. A self-assessment questionnaire to determine morningness-eveningness in human circadian rhythms. Int J Chronobiol 4, 97–110 (1976).

[13] Roenneberg T, Wirz-Justice A, Merrow M. Life between clocks: daily temporal patterns of human chronotypes. J Biol Rhythms. 2003 Feb;18(1):80-90.

[14] Ghotbi N., Pilz L.K., Winnebeck E., Vetter C., Zerbini G., Lenssen D., Frighetto G., Salamanca M., Costa R., Montagnese S., et al. The µMCTQ—An ultra-short version of the Munich ChronoType Questionnaire. in press. [Google Scholar]

[15] Terman J.S., Terman M., Lo E.S., Cooper T.B. Circadian time of morning light administration and therapeutic response in winter depression. Arch. Gen. Psychiatry Res· 2001;58:69–75.

---

[16] Roenneberg T, Pilz LK, Zerbini G, Winnebeck EC· Chronotype and Social Jetlag:

A (Self-) Critical Review. Biology (Basel). 2019 Jul 12;8(3). Review.

[17] https://www.gov.uk/government/publications/general-practice-physical-activity-questionnaire-gppaq

[18] Mackenzie IS, Rogers A, Poulter NR, Williams B, Brown MJ, Webb DJ, Ford I, Rorie DA, Guthrie G, Grieve JWK, Pigazzani F, Rothwell PM, Young R, McConnachie A, Struthers AD, Lang CC, MacDonald TM; TIME Study Group. Cardiovascular outcomes in adults with hypertension with evening versus morning dosing of usual antihypertensives in the UK (TIME study): a prospective, randomised, open-label, blinded-endpoint clinical trial. Lancet. 2022 Oct 22;400(10361):1417-1425.
